# Prof. Tardieu

**Published:** 1870-06

**Authors:** 


***Back Numbers Wanted.***

☞ Twenty-five cents, each, will be paid for Nos. 11, 12, 15 and 16 of this Journal, for 1869.

***The Transactions***

Take up so much space in this number, that we are reluctantly compelled to defer much valuable matter with which we have been favored. Meanwhile, we promise, if the American Medical Association does not sustain itself better the next year than for this and several years past, we shall take the liberty hereafter utterly to ignore its proceedings.

**Prof. Tardieu.**

[By the following clipping from a recent Paris letter, it will be seen that ours is not the only great capital where confusion has prevailed in Æsculapian circles.]

"The medical students are in revolt. One of their professors, Dr. Tardieu, is an expert of acknowledged ability. He was called in by Prince Pierre Napoleon to examine the wound on his jaw, as alleged to have been inflicted by Victor Noir, a few hours after the event at Auteuil. Before the High Court at Tours, the Doctor forgot the man of science in the courtier. The students took note of his partial evidence, and when he resumed his lectures he was received with hisses, hootings, cries of "resign," "go to the Tuileries," etc. This state of things has now existed for several days, becoming intensified by time. The amphitheatre, where the doctor lectures, can accommodate 1,000 persons, but 500 more squeeze in to see the sport. Four o'clock in the afternoon is the hour for lecture, but long before this the theatre is full. Some students kill time in reading novels, others arranging their bets on the result of the "demonstration" in hand, while the vast majority fall back on music. The concert opens with the popular chant, "Holy Spirit, descend upon us," which would be unobjectionable if it were not so immediately followed by cheers for Patti, and "*la femme a barbe*," no allusion *la diva*. The only orchestral accompaniments are wooden and tin whistles. There is a sudden and combined howl; all eyes are turned upward, and some half dozen students are observed on the glass roof, grinning like gorgons at those below. Music has its charms, for the *Marseillaise* is sung, and the performers, pleased with their rendering of it, cheer their noble selves and resume the refrain. Vivas follow for Victor Noir and Rochfort, the latter being at last voted President of the French Republic, with full powers to give the students everything they may demand. It is four o'clock; the door opens; quite a stream of doctors enter, and at last Tardieu, who, pale and grave mounts the estrade—Pandemonium then breaks loose—the doctor bows and retires, followed to his carriage by the Furies. But a number of strangers have got into the crowd—above all, law students, with whom the hunting down of a medical expert ranks among the first of legal virtues. But Bob Sawyer is just—he will have none but Athenians proper, to pronounce an ostracism, and for the future, every medical student must present his card before being admitted to the lecture-room. Thus a fair vote will be taken on Dr. Tardieu. The pupils—especially the juniors—called the "Mountain"—demand his resignation. This he refuses, and the other professors promise to make common cause with him; if so, the University of Medicine must be temporarily closed. In 1865, Dr. Tardieu resigned as Dean of the University, rather than communicate what he considered an unmerited reprimand from the Minister of Public Instruction to the students. But of late, his evidence before juries was more of the advocate than the cool impartiality of the judge."

